# A multi-taxon analysis of European Red Lists reveals major threats to biodiversity

**DOI:** 10.1371/journal.pone.0293083

**Published:** 2023-11-08

**Authors:** Axel Hochkirch, Melanie Bilz, Catarina C. Ferreira, Anja Danielczak, David Allen, Ana Nieto, Carlo Rondinini, Kate Harding, Craig Hilton-Taylor, Caroline M. Pollock, Mary Seddon, Jean-Christophe Vié, Keith N.A. Alexander, Emily Beech, Manuel Biscoito, Yoan Braud, Ian J. Burfield, Filippo Maria Buzzetti, Marta Cálix, Kent E. Carpenter, Ning Labbish Chao, Dragan Chobanov, Maarten J. M. Christenhusz, Bruce B. Collette, Mia T. Comeros-Raynal, Neil Cox, Matthew Craig, Annabelle Cuttelod, William R. T. Darwall, Benoit Dodelin, Nicholas K. Dulvy, Eve Englefield, Michael F. Fay, Nicholas Fettes, Jörg Freyhof, Silvia García, Mariana García Criado, Michael Harvey, Nick Hodgetts, Christina Ieronymidou, Vincent J. Kalkman, Shelagh P. Kell, James Kemp, Sonia Khela, Richard V. Lansdown, Julia M. Lawson, Danna J. Leaman, Joana Magos Brehm, Nigel Maxted, Rebecca M. Miller, Eike Neubert, Baudewijn Odé, David Pollard, Riley Pollom, Rob Pople, Juan José Presa Asensio, Gina M. Ralph, Hassan Rankou, Malin Rivers, Stuart P. M. Roberts, Barry Russell, Alexander Sennikov, Fabien Soldati, Anna Staneva, Emilie Stump, Andy Symes, Dmitry Telnov, Helen Temple, Andrew Terry, Anastasiya Timoshyna, Chris van Swaay, Henry Väre, Rachel H. L. Walls, Luc Willemse, Brett Wilson, Jemma Window, Emma G. E. Wright, Thomas Zuna-Kratky

**Affiliations:** 1 Musée National d’Histoire Naturelle, Luxembourg, Luxembourg; 2 Department of Biogeography, Trier University, Trier, Germany; 3 IUCN SSC Invertebrate Conservation Committee, Trier, Germany; 4 IUCN SSC Steering Committee, Caracas, Venezuela; 5 IUCN SSC Grasshopper Specialist Group, Trier, Germany; 6 Institute of Landscape Architecture and Environmental Planning, Technische Universität Berlin, Berlin, Germany; 7 IUCN SSC Freshwater Plant Specialist Group, Stroud, United Kingdom; 8 IUCN European Regional Office, Brussels, Belgium; 9 UFZ—Helmholtz Centre for Environmental Research, Department of Conservation Biology, Leipzig, Germany; 10 IUCN, Biodiversity Assessment and Knowledge Team, Cambridge, United Kingdom; 11 IUCN, Species Conservation Action Team, Gland, Switzerland; 12 Global Mammal Assessment program, Department of Biology and Biotechnologies, Sapienza University of Rome; Rome, Italy; 13 Global Wildlife Conservation Center, State University of New York College of Environmental Science and Forestry, Syracuse, NY, United States of America; 14 IUCN SSC Mollusc Specialist Group, Devon, United Kingdom; 15 Fondation Franklinia, Genève, Switzerland; 16 IUCN SSC Plant Conservation Committee, Pretoria, South Africa; 17 IUCN Specialist Adviser on European Saproxylic Beetles, Truro, United Kingdom; 18 Botanic Gardens Conservation International, Richmond, United Kingdom; 19 Funchal Natural History Museum, Funchal, Portugal; 20 MARE-Marine and Environmental Sciences Centre, Lisboa, Portugal; 21 BirdLife International, Cambridge, United Kingdom; 22 IUCN SSC Red List Authority for Birds, Cambridge, United Kingdom; 23 Fondazione Museo Civico di Rovereto, Sezione Zoologia, Rovereto, Italy; 24 Rewilding Portugal, Guarda, Portugal; 25 IUCN Marine Biodiversity Unit, Biological Sciences, Norfolk, VA, United States of America; 26 National Museum of Marine Biology, Checheng, Taiwan; 27 Institute of Biodiversity and Ecosystem Research, Bulgarian Academy of Sciences, Sofia, Bulgaria; 28 Plant Gateway Herbarium, Kingston upon Thames, United Kingdom; 29 IUCN Tuna and Billfish Specialist Group, National Museum of Natural History, Washington, DC, United States of America; 30 ARC Centre of Excellence for Coral Reef Studies, James Cook University, Townsville, Australia; 31 Water Resources Research Center, University of Hawai’i, Honolulu, HI, United States of America; 32 IUCN-Conservation International Biodiversity Assessment Unit, Washington, DC, United States of America; 33 National Oceanic and Atmospheric Administration, National Marine Fisheries Service, Southwest Fisheries Science Center, La Jolla, CA, United States of America; 34 IUCN Red List Unit, IUCN Global Species Programme, Cambridge, United Kingdom; 35 Earth to Ocean Research Group, Department of Biological Sciences, Simon Fraser University, Burnaby, Canada; 36 Joint Nature Conservation Committee, Peterborough, United Kingdom; 37 IUCN SSC Orchid Specialist Group, Royal Botanic Gardens; Richmond, United Kingdom; 38 Scott Cawley, Dublin, Ireland; 39 Museum für Naturkunde, Leibniz Institute for Evolution and Biodiversity Science, Berlin, Germany; 40 Oceana, Madrid, Spain; 41 School of Geosciences, The University of Edinburgh, Edinburgh, United Kingdom; 42 European Committee for the Conservation of Bryophytes, Portree, United Kingdom; 43 BirdLife Cyprus, Nikosia, Cyprus; 44 Naturalis Biodiversity Center, Leiden, The Netherlands; 45 The University of Birmingham, School of Biosciences, Birmingham, United Kingdom; 46 IUCN SSC Cave Invertebrate Specialist Group, Cambridge, United Kingdom; 47 Bren School of Environmental Science & Management, University of California, Santa Barbara, Santa Barbara, CA, United States of America; 48 IUCN SSC Medicinal Plant Specialist Group, Ottawa, Canada; 49 IUCN SSC Crop Wild Relative Specialist Group, Birmingham, United Kingdom; 50 Natural History Museum Bern, Bern, Switzerland; 51 FLORON Plant Conservation Netherlands, Nijmegen, Netherlands; 52 Department of Ichthyology, Australian Museum, Sydney, Australia; 53 Species Recovery Program, Seattle Aquarium, Seattle, WA, United States of America; 54 Departamento de Zoología, Facultad de Biología, Universidad de Murcia; Murcia, España; 55 IUCN SSC Global Tree Specialist Group, Richmond, United Kingdom; 56 Department of Agroecology, Université Libre de Bruxelles (ULB), Brussels, Belgium; 57 IUCN Snapper, Seabream and Grunt Specialist Group, Museum and Art Gallery of the Northern Territory, Darwin, Australia; 58 Botanical Museum, Finnish Museum of Natural History, University of Helsinki, Helsinki, Finland; 59 Office National des Forêts, Laboratoire National d’Entomologie Forestière, Quillan, France; 60 Natural History Museum, Department of Life Sciences, London, United Kingdom; 61 Coleopterological Research Center, Institute of Life Sciences and Technology, Daugavpils University, Daugavpils, Latvia; 62 Institute of Biology, University of Latvia, Rīga, Latvia; 63 The Biodiversity Consultancy, Cambridge, United Kingdom; 64 Zoological Society of London, London, United Kingdom; 65 TRAFFIC, Cambridge, United Kingdom; 66 Vlinderstichting (Dutch Butterfly Conservation), Wageningen, Netherlands; 67 Reef Environmental Education Foundation, Key Largo, FL, United States of America; 68 Department of Plant Sciences, University of Cambridge, Cambridge, United Kingdom; 69 Ingenieurbüro für Landschaftsplanung und Landschaftspflege, Vienna, Austria; Shiv Nadar University, INDIA

## Abstract

Biodiversity loss is a major global challenge and minimizing extinction rates is the goal of several multilateral environmental agreements. Policy decisions require comprehensive, spatially explicit information on species’ distributions and threats. We present an analysis of the conservation status of 14,669 European terrestrial, freshwater and marine species (ca. 10% of the continental fauna and flora), including all vertebrates and selected groups of invertebrates and plants. Our results reveal that 19% of European species are threatened with extinction, with higher extinction risks for plants (27%) and invertebrates (24%) compared to vertebrates (18%). These numbers exceed recent IPBES (Intergovernmental Platform on Biodiversity and Ecosystem Services) assumptions of extinction risk. Changes in agricultural practices and associated habitat loss, overharvesting, pollution and development are major threats to biodiversity. Maintaining and restoring sustainable land and water use practices is crucial to minimize future biodiversity declines.

## Introduction

Biodiversity is declining globally at an unprecedented rate [1–3], with around 1 million animal, fungal and plant species potentially at risk of extinction within the next few decades [4]. Several international policies have been designed to tackle this crisis, namely by defining specific biodiversity recovery goals and targets (e.g., the United Nations Sustainable Development Goals (SDG 14, 15), the Convention on Biological Diversity (CBD) Aichi Targets and Kunming-Montreal Global Biodiversity Framework Targets) that have been transposed into national or regional policy by countries worldwide. To document progress towards these targets spatially explicit information on the distribution of species, their ecological requirements and major threats is needed [5, 6]. Red List assessments that compile the best available evidence on species’ extinction risk are pivotal to measure progress towards international biodiversity conservation objectives by underpinning suitable biodiversity indicators [7]. The IUCN Red List of Threatened Species^TM^ (hereafter, the IUCN Red List) is widely recognized as the most comprehensive and objective approach for evaluating the conservation status of species, and is considered a global ‘barometer of life’ [8]. More than 142,000 species have been assessed for the IUCN Red List thus far, but at the global scale there are strong taxonomic biases [6].

In Europe, taxonomic coverage of the IUCN Red List is more extensive than in other parts of the world, as the European Commission has funded European Red List assessments of thousands of species from a wide variety of taxonomic groups since 2006. These include all vertebrates (amphibians, birds, fishes, mammals and reptiles), functionally important invertebrate groups (all bees, butterflies, dragonflies, grasshoppers, crickets, bush-crickets, freshwater and terrestrial molluscs, and a selection of saproxylic beetles) and about 12% of the known plant species in Europe (including all ferns and lycopods, orchids, trees, aquatic plants and bryophytes, as well as selected shrubs, medicinal plants, priority crop wild relatives, and plants listed in policy instruments). This Herculean effort provides a wealth of information on the conservation status of 14,669 species, including spatial information on an exceptionally broad range of species that is derived using a standardized methodology and includes taxa that are usually underrepresented in conservation [6]. The assessed taxa have not been chosen to ensure representativeness but based upon funders’ priorities. However, they are by far more diverse than any dataset used for global analyses so far, such as the Living Planet Index [9]. These data will help to guide and monitor progress in achieving the targets of the EU Biodiversity Strategy for 2030 [10], i.e., to ensure that Europe’s biodiversity is on the path to recovery by 2030. Here, we synthesize the findings of all European Red List species assessments published up to the end of 2020 to analyze major biodiversity distribution patterns and threats to biodiversity in Europe. This analysis also provides a baseline against which to measure progress towards biodiversity targets to be achieved in the coming decade.

### Results

**Fig 1:**
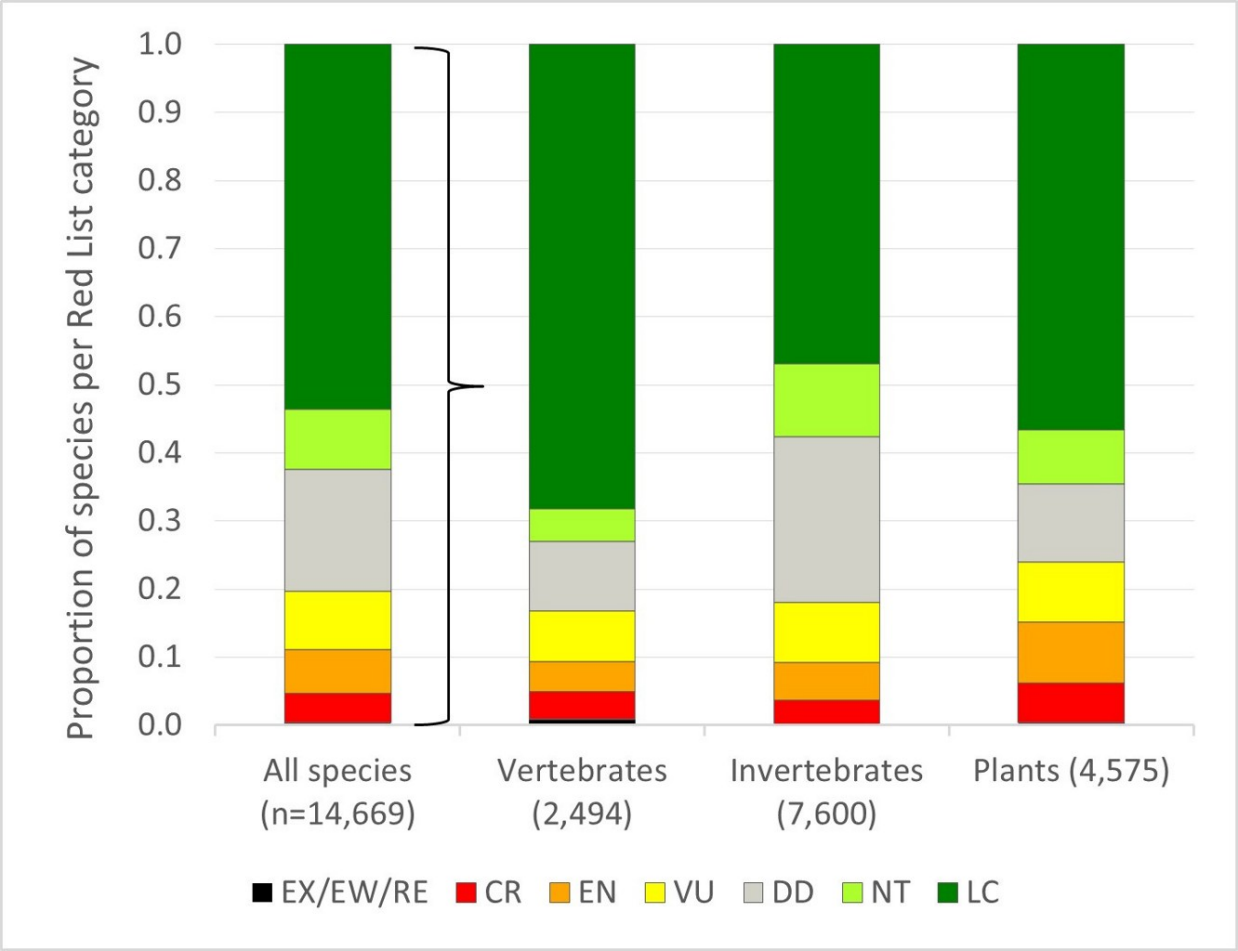
IUCN Red List status of 14,669 European species. Abbreviations: EX: Extinct, EW: Extinct in the Wild, RE: Regionally Extinct, CR: Critically Endangered, EN: Endangered, VU: Vulnerable, DD: Data Deficient, NT: Near Threatened, LC: Least Concern.

In Europe, approximately one-fifth (19.4%, 2,839 species) of the 14,669 species assessed are threatened with extinction (Fig 1) with 50 species being Extinct, Regionally Extinct or Extinct in the Wild (EX, RE, EW) and a further 75 tagged as Possibly Extinct. The percentage of threatened species (those classified as Critically Endangered (CR), Endangered (EN) or Vulnerable (VU)) was higher among plants (27%) and invertebrates (24%) than among vertebrates (18%). This pattern is noteworthy considering that vertebrates receive substantially more conservation attention and that the latest IPBES (Intergovernmental Science-Policy Platform on Biodiversity and Ecosystem Services) global assessment on biodiversity and ecosystem services used a conservative “tentative estimate” that 10% of all insects are threatened with extinction, while noting that “*the prevalence of extinction risk in high-diversity insect groups is a key unknown*” [4]. Using our value of 24% threatened invertebrates, would roughly double the IPBES extrapolation (1.97 ± 0.23 million species threatened rather than 1 million). It is worth noting that IPBES also used the European Red Lists for bees, butterflies and saproxylic beetles to estimate the global extinction risk of insects. While the extrapolation of European data to a global estimate involves several uncertainties, evidence from some comprehensively assessed species groups suggests that global extinction risk does not deviate strongly from the European status (e.g. Odonata: European Red List [11]: 15.7% threatened, Global Red List [12]: 16.1% threatened; Birds: European Red List [13]: 13.2%, Global Red List [11]: 12.6%). Our higher assumption of the number of threatened insect species is mainly explained by the inclusion of recent European Red Lists compared to the IPBES assessment, and partly by the high number of Data Deficient (DD) species among insects (S2 Fig). Indeed, the number of DD species is quite high even in Europe (18%), despite this being a very well-studied region. Data deficiency is notably higher among invertebrates (24%) than plants (11%) or vertebrates (10%). Further, for nearly half of all species (49%) and for 60% of invertebrates, the population trend was classified as ‘unknown’ by the Red List assessors, which is in line with global estimates and illustrates a general lack of data on population status and demographics and confirms the need for biodiversity monitoring programs [6].

**Fig 2:**
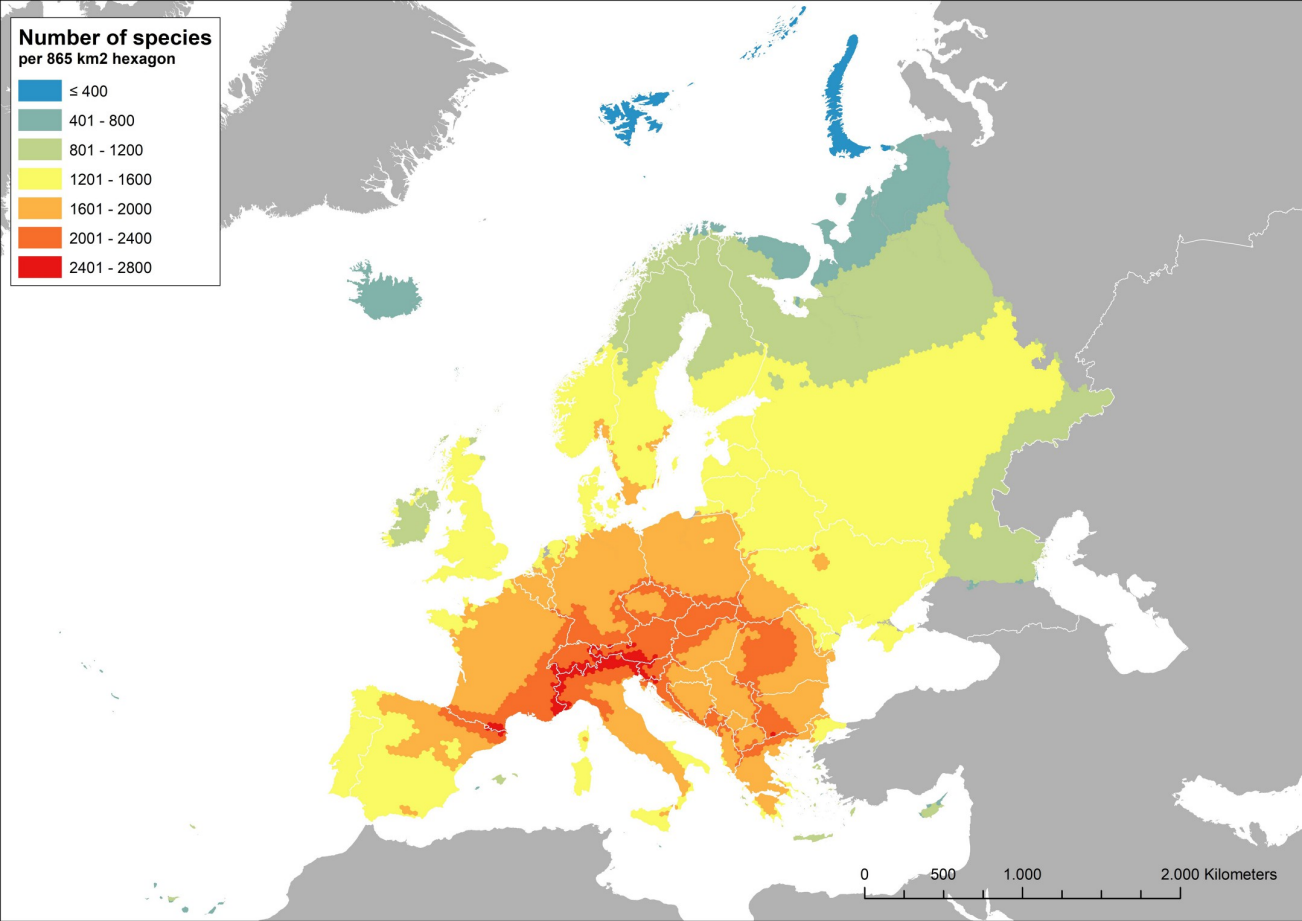
Species richness in Europe. Spatial distribution of terrestrial and freshwater species richness in Europe based on an analysis of all European IUCN Red List assessments.

Nearly half (47%, *n* = 6,926 of the 14,669) of Europe’s assessed species are endemic, including 2,125 threatened species. Most (86%, n = 1,171) threatened invertebrates are endemic to Europe. Across all taxa, only half (54%) of the threatened species have been documented in protected areas, a percentage lower than among Near Threatened (NT) or Least Concern (LC) species (61%), raising concerns about the suitability of the European protected area network as a means to protect all threatened species [14, 15] and emphasizing the need to expand and improve it. Our spatial analysis of terrestrial species diversity in Europe (Fig 2) further emphasizes the importance of mountain systems for biodiversity persistence in Europe. Mountains support a high number of endemic species and are also less transformed by humans than lowland plains and coasts. The highest species numbers by area were recorded in the southern Alps, the eastern Pyrenees and the Pirin Mountains in Bulgaria (Fig 2), while threatened biodiversity peaks in the Alps and the Balkans (S5 Fig).

**Fig 3:**
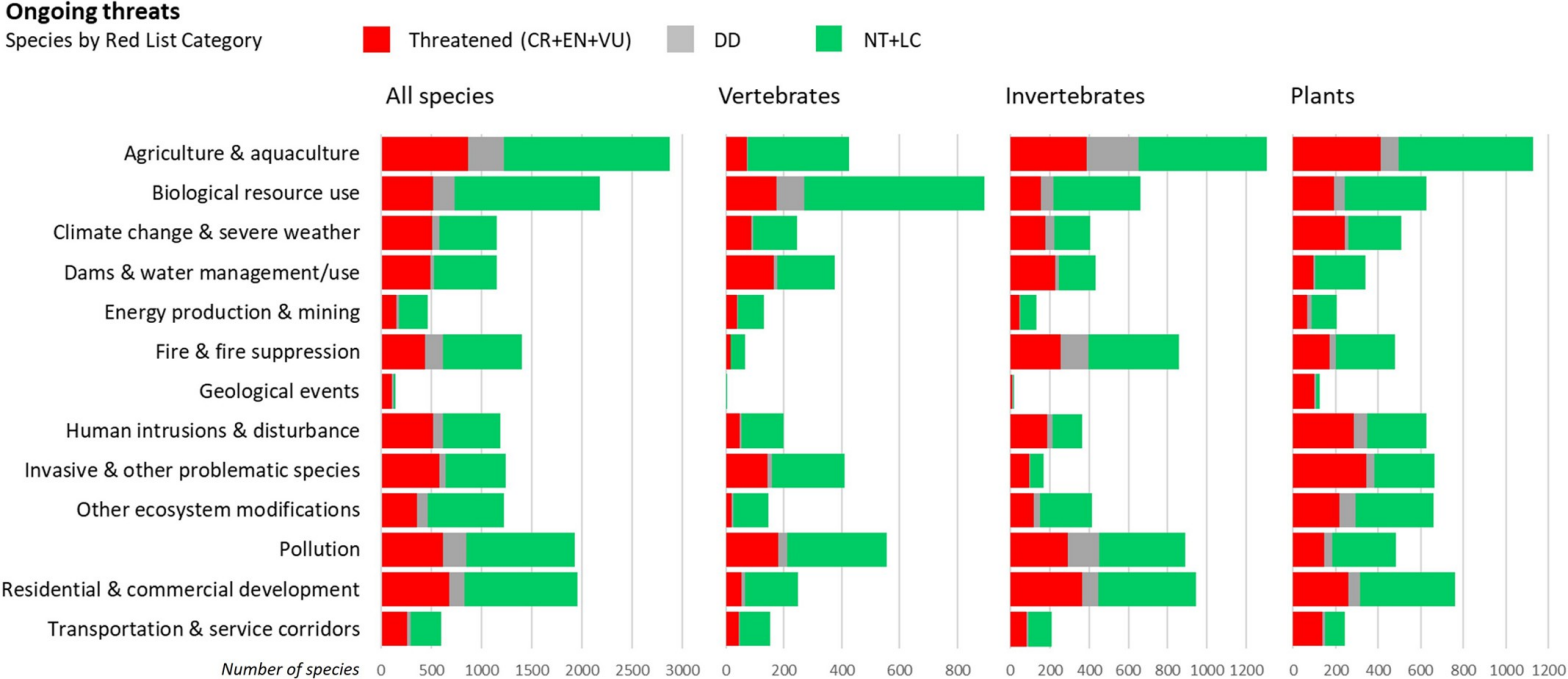
Major threats to biodiversity in Europe. For all species, vertebrates, invertebrates and plants (CR: Critically Endangered, EN: Endangered, VU: Vulnerable, DD: Data Deficient, NT: Near Threatened, LC: Least Concern; N: All species = 14,669, Vertebrates = 2,494, Invertebrates = 7,600, Plants = 4,575).

Our analyses confirm that multiple threats impact biodiversity, with agricultural land-use change (including tree plantations) being the most important threat to European species, followed by biological resource use (overexploitation), residential and commercial development, and pollution (Fig 3). The strong impact of agricultural land-use is more prominent in invertebrates and plants, whereas vertebrates (particularly fishes) are more often threatened by overexploitation as they may be directly hunted, caught and fished (also by incidental catch) resulting in extensive threat to marine fishes and other marine vertebrates. Residential and commercial development is an important cause of habitat loss and degradation affecting many invertebrate and plant species, whereas pollution is particularly threatening to freshwater species, such as fishes, molluscs and dragonflies. Climate change is also an important threat to many species and has been classified as the most important emerging future threat (S3 Fig). This is corroborated by the increasing number of droughts in Europe, which accelerate the risk of wildfires [16], aggravated by an increased off-take of water for agriculture and domestic supplies.

## Discussion

The finding of agricultural land-use change as a major threat to biodiversity has often been reported [e.g. 17, 18]. However, our analysis is the most comprehensive and unequivocal to date reaffirming the magnitude of the impact of this threat at a continental scale. Many European species require or are adapted to traditional agricultural land-use but cannot cope with the magnitude of this change. Changes in agriculture are manifold and include conversion of natural habitats into farmland (partly as a consequence of detrimental subsidies under the EU Common Agricultural Policy (CAP)), changing agricultural and forestry practices (particularly intensification and homogenization of land-use with larger plots, larger and heavier machines, use of fertilizers and pesticides, decreasing crop diversity, higher livestock densities, earlier and more frequent mowing, drainage, irrigation, plowing, rolling, abandonment of historical management techniques, etc.), but also land abandonment coupled with rural exodus [19]. In Europe, habitat conversion into arable land mainly occurred in the past, while during the last decades abandonment has become more common. Intensification in the use of agricultural land had started already in the 19^th^ century in northwestern Europe with the replacement of traditional pastoral farming (mainly of sheep) by settled agriculture with cattle farming [20]. While pastoral systems are still abundant in the Mediterranean, they are also in decline due to the EU CAP funding systems [21]. While improvements to the CAP have constantly been proposed [22], the recent policy reform remained rather unambitious in this regard despite the promising wind of change brought by the European Green Deal. Most importantly, direct payments under the CAP have favored larger farms, while smallholder farming is in decline, leading to the abandonment of marginal lands, which are often particularly species-rich and reliant on extensive agricultural land-use [23]. While agricultural intensification is sometimes proposed as a means to increase the amount of natural habitats (“land sparing”), many threatened species in Europe are adapted to grassland habitats, which can only be retained by livestock grazing or mowing. Maintaining such habitat types will be challenging as traditional agricultural management is often not profitable anymore. Abandonment of traditional land use is also a threat to some forest species, which may depend on historical management such as coppicing or forest pastures.

Moreover, our analysis highlights some major knowledge gaps and research needs (S4 Fig). For a quarter of invertebrate species, the evidence available was not sufficient to determine their conservation status—most notably, 57% of European bees were assessed as Data Deficient [24]. Half of all species lack population trend data, which is a key requirement for assessing species extinction risk. This also means that for many species, Red List assessments are based on habitat trend information or other proxies. Unsurprisingly, the top research priorities identified for most species by the assessors include research on distribution, population sizes and trends, threats, life history and ecology as well as taxonomy (S4 Fig). Monitoring of population trends is also needed for many species, particularly for threatened taxa. In this context, it is important to highlight that general biodiversity monitoring schemes are usually not suitable for monitoring the status of highly threatened taxa (as these species are too rarely recorded to enable an analysis of trends). This means that targeted monitoring programs are required for species with a high extinction risk [25]. For vertebrate species, the need for research on the effectiveness of conservation actions has been identified more often than for plants or invertebrates. This could reflect a higher number of ongoing conservation projects for vertebrates compared to other taxa, which still require basic data to improve conservation assessments or compile conservation plans. While Europe probably has the most comprehensive Red List information in terms of species groups covered compared to other continents, the status of some key groups is still unexplored, such as freshwater quality indicators (e.g. mayflies, stoneflies, caddisflies), soil biota (e.g. fungi, springtails, earthworms, mites), decomposers (e.g. dung beetles, carrion beetles), marine invertebrates (e.g. marine crustaceans and mollusks), species-rich insect groups (e.g. weevils, rove beetles, leaf beetles, ground beetles) and many plant taxa. However, European Red List assessments have just been completed for hoverflies, are currently underway for moths, and a substantial portion of the taxa analyzed here are undergoing a reassessment which will lead to the development of Red List indices. Hence, the taxonomic and temporal coverage of the European Red Lists is constantly being increased.

Red Lists provide a valuable baseline for measuring progress towards biodiversity targets. Due to their wide taxonomic scope, the European Red Lists have revealed high extinction risks for some taxa, such as freshwater molluscs (59% threatened, [26]), trees (42%, [27]), freshwater fishes (40%, [28]) and Orthoptera (29%, [29]). As biodiversity recovery targets have become more refined under the Kunming-Montréal Global Biodiversity Framework, it will be important to continue to take snapshots of the biodiversity status not only in Europe, but at a global scale. To that end, metrics derived from the Red Lists, such as the Red List Index, have been adopted as indicators to track progress on meeting international conservation policy commitments and Sustainable Development Goals [7, 30].

While the measurement and assessment of biodiversity trends is crucial to guide policy, it is even more important to implement necessary conservation action in a timely manner. We already have enough evidence at hand to act—what we are missing is action. This requires collaboration among multiple stakeholders to abate the major threats identified [31]. Indeed, conservation NGOs, conservation authorities, species experts and citizens in Europe have started numerous projects, focusing on highly threatened species, and even including threatened invertebrates, as a consequence of Red List publications [32–35]. Funding mechanisms for implementing conservation action exist at the European level (e.g. EU LIFE program) as well as on an international, national or even local scale. Member States now need to increase their capacity to conduct or support conservation projects and create optimal structures to plan and implement conservation action. Furthermore, biodiversity conservation needs to be better integrated or mainstreamed within other policies, so that the impact of major threats (such as agriculture, overfishing, forestry, pollution, urban and rural development) is mitigated. So far, financial investment in activities detrimental to biodiversity far outstrips biodiversity-friendly investments [36, 37]. Biodiversity is the foundation underpinning food security, human well-being and wealth generation and securing a future for European life requires greener agriculture and fishing policies and a rapid phasing out of incentives detrimental to biodiversity in agriculture, forestry, fisheries and energy production are needed.

## Materials and methods

All European Red Lists published to date can be found at http://ec.europa.eu/environment/nature/conservation/species/redlist/.

The following Red Lists were considered for the analyses: European Red List of amphibians [38], European Red List of birds [13], European Red List of freshwater fishes [28], European Red List of marine fishes [39], European Red List of mammals [40], European Red List of reptiles [41], European Red List of bees [24], European Red List of saproxylic beetles [42, 43], European Red List of butterflies [44], European Red List of dragonflies [11], European Red List of non-marine molluscs [26], European Red List of terrestrial molluscs [45], European Red List of grasshoppers, crickets and bush-crickets [29], European Red List of vascular plants [46], European Red List of medicinal plants [47], European Red List of trees [27], European Red List of lycopods and ferns [48], European Red List of mosses, liverworts and hornworts [49].

The European Red List operates at the geographical scope of Europe extending to the Urals in the east, and from Franz Josef Land in the north to the Mediterranean in the south (S1 Fig). The Canary Islands, Madeira and the Azores are also included. In the southeast, the Caucasus region is not included in most assessments, except for the bird assessments, which also cover Turkey, the Caucasus region, and Greenland [13]. For the boundaries of marine assessments see S1 Fig. The European Red Lists were compiled using the IUCN Red List Categories and Criteria at regional level [50]. All species were assessed against the IUCN Red List Criteria to assess their extinction risk and categorized into nine categories [51] at the regional scale: Data Deficient (DD), Least Concern (LC), Near Threatened (NT), Vulnerable (VU), Endangered (EN), Critically Endangered (CR), Regionally Extinct (RE), Extinct in the Wild (EW), Extinct (EX). These categories are defined in the IUCN guidelines for application of IUCN Red List criteria at regional and national levels [50]. The terms RE and EW are sometimes referred to as “regionally extirpated” or “extirpated in the wild”, but we follow the IUCN definition here, which is widely used in the scientific literature. Species classified as CR, EN, or VU are considered threatened with extinction. Each assessment is supported, where available, by information on distribution (including a range map), population, ecology, threats, as well as necessary or existing conservation action and research. This information is provided as free text, but also collected in standardized classification schemes (following the standard system provided by [52]), which were analyzed here to obtain European distribution, threat and research information across taxa. Species presence in protected areas was also recorded (as presence in protected areas yes/no).

All analyses (Red List categories and totals by classification field) were carried out for the set of all species as well as for vertebrates, invertebrates and plants separately. To account for changes in the assessments since 2006, an updated dataset was created from the IUCN Red List version 2019–2. The percentage of threatened species was calculated as the “best estimate” as recommended by IUCN [53]: EW + CR + EN + VU / (total assessed—EX—DD). This method assumes that a similar relative percentage of the Data Deficient (DD) species are likely to be threatened. All following analyses considered only species extant in the wild (i.e. excluding species categorized as EX, EW and RE). The ongoing and future threats recorded for extant species were analyzed based upon the classification schemes in the IUCN Red List. The highest threat level category was used [52], except for category 7 ‘natural system modifications’, where the second level was analyzed (i.e. ‘Fire & fire suppression’, ‘Dams & water management/use’ and ‘Other ecosystem modifications’).

For each species, assessors were asked to produce the most accurate depiction of a taxon’s current and historical distribution based on their knowledge and the available data. Data sources informing the production of range maps have changed over the various European Red Lists as a result of the increasing availability of digitized georeferenced locality record data (e.g. Global Biodiversity Information Facility (GBIF), frequently viewed through the Geospatial Conservation Assessment Tool (GeoCAT) which was launched in 2011 [54]. The general approach has been for assessors to compile and review available locality records for a taxon, and then produce polygons that encompass the known (locality records) and inferred (based on ecological requirements of the taxon) range of the taxon. Freshwater taxa (fishes, molluscs, Odonata, aquatic plants) were mapped to river sub-catchments (HydroBASINS or earlier iterations). All distribution maps were produced as polygon GIS shapefiles in WGS 1984 (World Geodetic Survey 1984 projection; see [55] for metadata requirements). For detailed mapping methodology, see the individual European Red List reports. The species richness maps presented in this publication were analyzed using a geodesic discrete global grid system, defined on an icosahedron and projected to the sphere using the inverse Icosahedral Snyder Equal Area (ISEA) Projection (S39). This corresponds to a hexagonal grid composed of individual units (cells) that retain their shape and area (864 km²) throughout the globe. For the spatial analyses, only the extant (resident) and possibly extant (resident) distributions of each species were converted to the hexagonal grid; polygons coded as ‘possibly extinct’, ‘extinct’, ‘re-introduced’, ‘introduced’, ‘vagrant’ and/or ‘presence uncertain’ were not considered in the analyses. Coastal cells were clipped to the coastline. Thus, patterns of species richness were mapped by counting the number of species in each cell (or cell section, for species with a coastal distribution). Data Deficient species and species that were only mapped to country-level were excluded from the analysis. Patterns of threatened species richness (Categories CR, EN, VU) were mapped by counting the number of threatened species in each cell or cell section.

## Supporting information

S1 Fig Spatial extent of European Red List assessments for most terrestrial and freshwater taxa (orange), marine mammals (light blue) and marine fishes (dark blue). (PNG)

S2 Fig IUCN Red List Categories and number of species assessed for Europe by taxonomic group (groups marked with * have not been assessed comprehensively; black lines indicate the best estimate for the proportion of extant species considered to be threatened). Seven mollusc species have been classed as both freshwater and terrestrial and are listed in both groups.
(JPG)

S3 Fig Emerging future threats to biodiversity in Europe for all species, and for vertebrates, invertebrates and plants separately (CR: Critically Endangered, EN: Endangered, VU: Vulnerable, DD: Data Deficient, NT: Near Threatened, LC: Least Concern; N: All species = 14,669, Vertebrates = 2,494, Invertebrates = 7,600, Plants = 4,575). (JPG)

S4 Fig Major research needs in Europe as classified by the Red List assessors for all species, and for vertebrates, invertebrates and plants separately (CR: Critically Endangered, EN: Endangered, VU: Vulnerable, DD: Data Deficient, NT: Near Threatened, LC: Least Concern; N: All species = 14,669, Vertebrates = 2,494, Invertebrates = 7,600, Plants = 4,575). (JPG)

S5 Fig Number of threatened terrestrial and freshwater species across Europe (i.e. Red List categories CR, EN, VU). (JPG)
