# Supplementary figures and images for: A multi-taxon analysis of European Red Lists reveals major threats to biodiversity
Source: PLoS One. 2023 Nov 8;18(11):e0293083. doi: 10.1371/journal.pone.0293083 (PMC10631624; doi:10.1371/journal.pone.0293083)

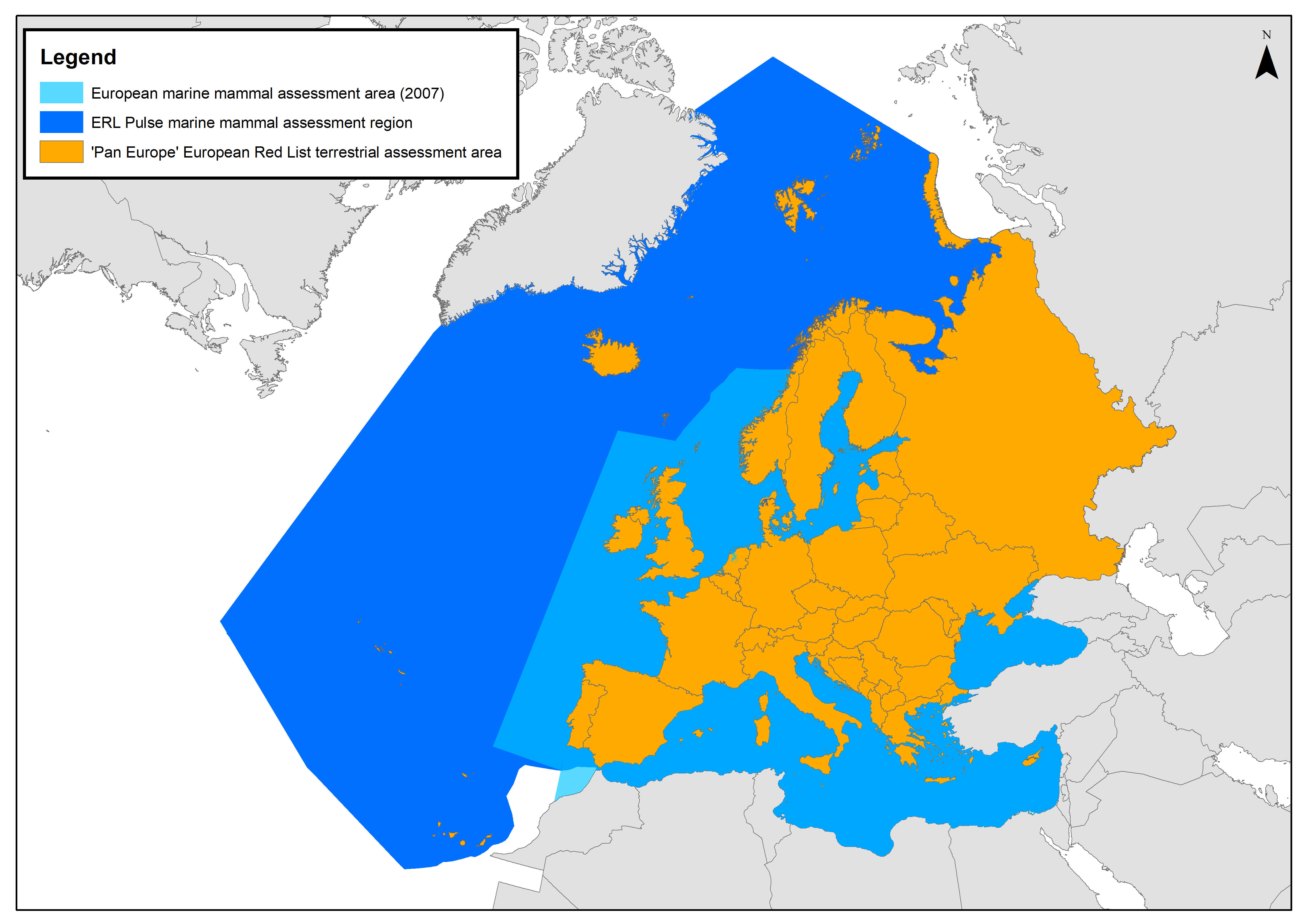

Supplement: S1 Fig — (PNG) [file pone.0293083.s001.png]

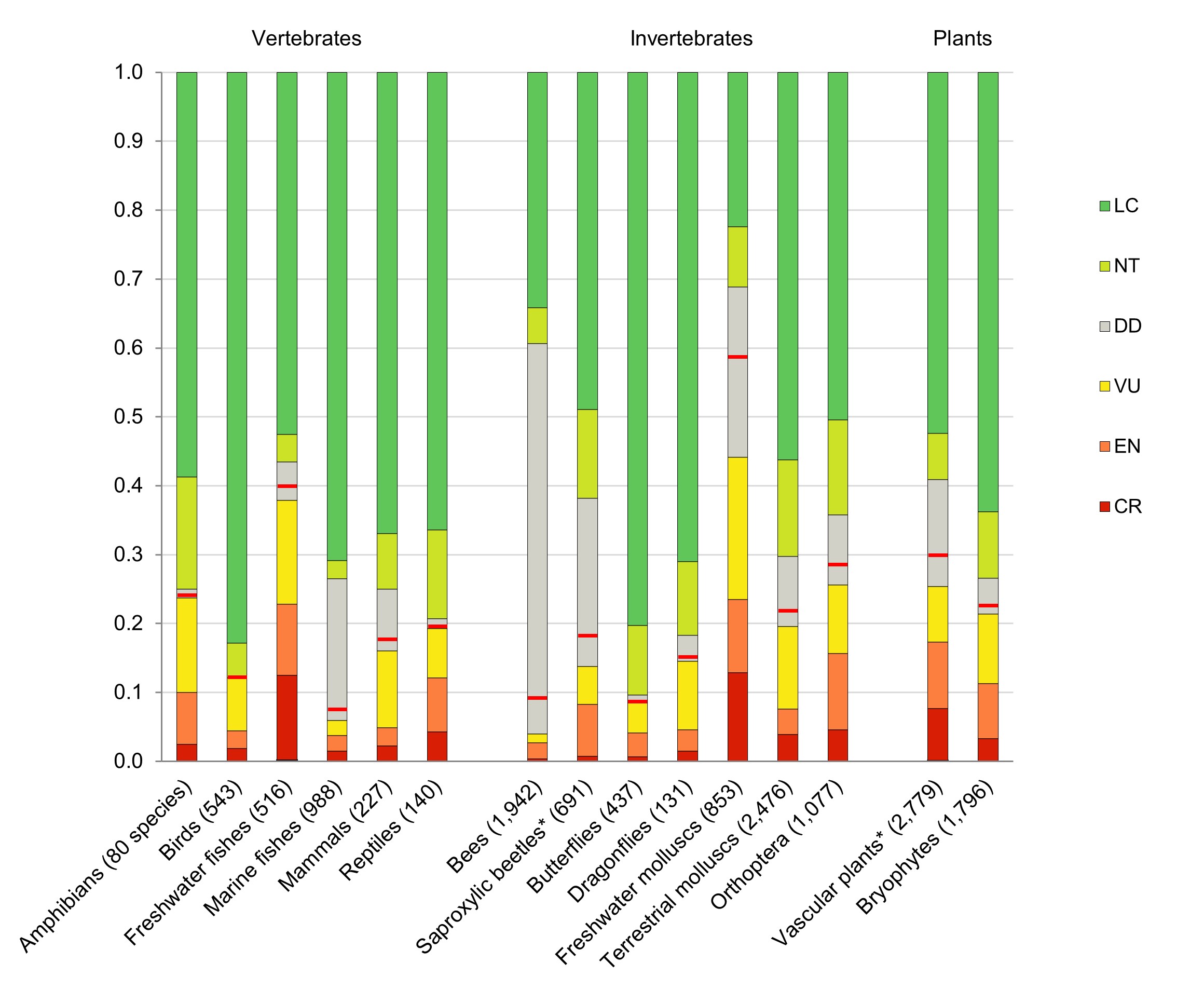

Supplement: S2 Fig — Seven mollusc species have been classed as both freshwater and terrestrial and are listed in both groups. (JPG) [file pone.0293083.s002.jpg]

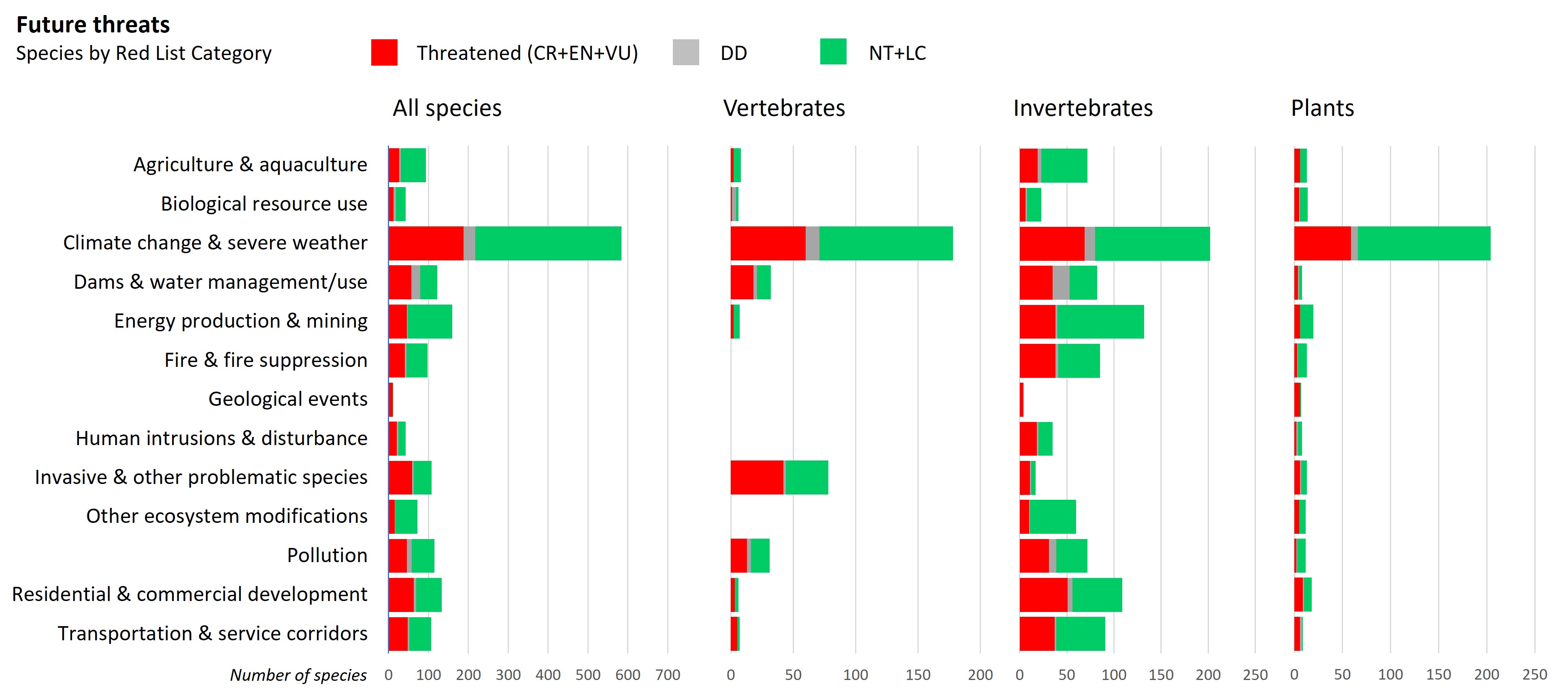

Supplement: S3 Fig — (JPG) [file pone.0293083.s003.jpg]

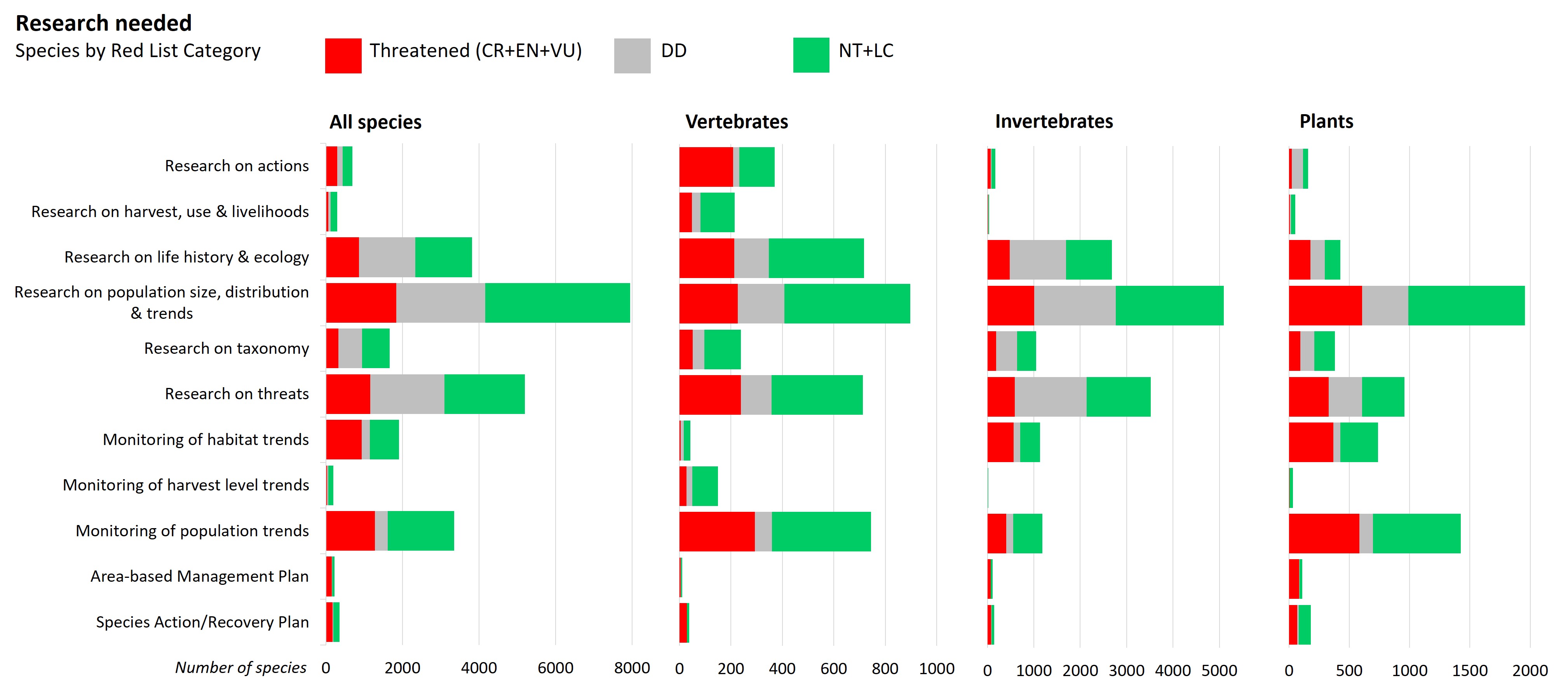

Supplement: S4 Fig — (JPG) [file pone.0293083.s004.jpg]

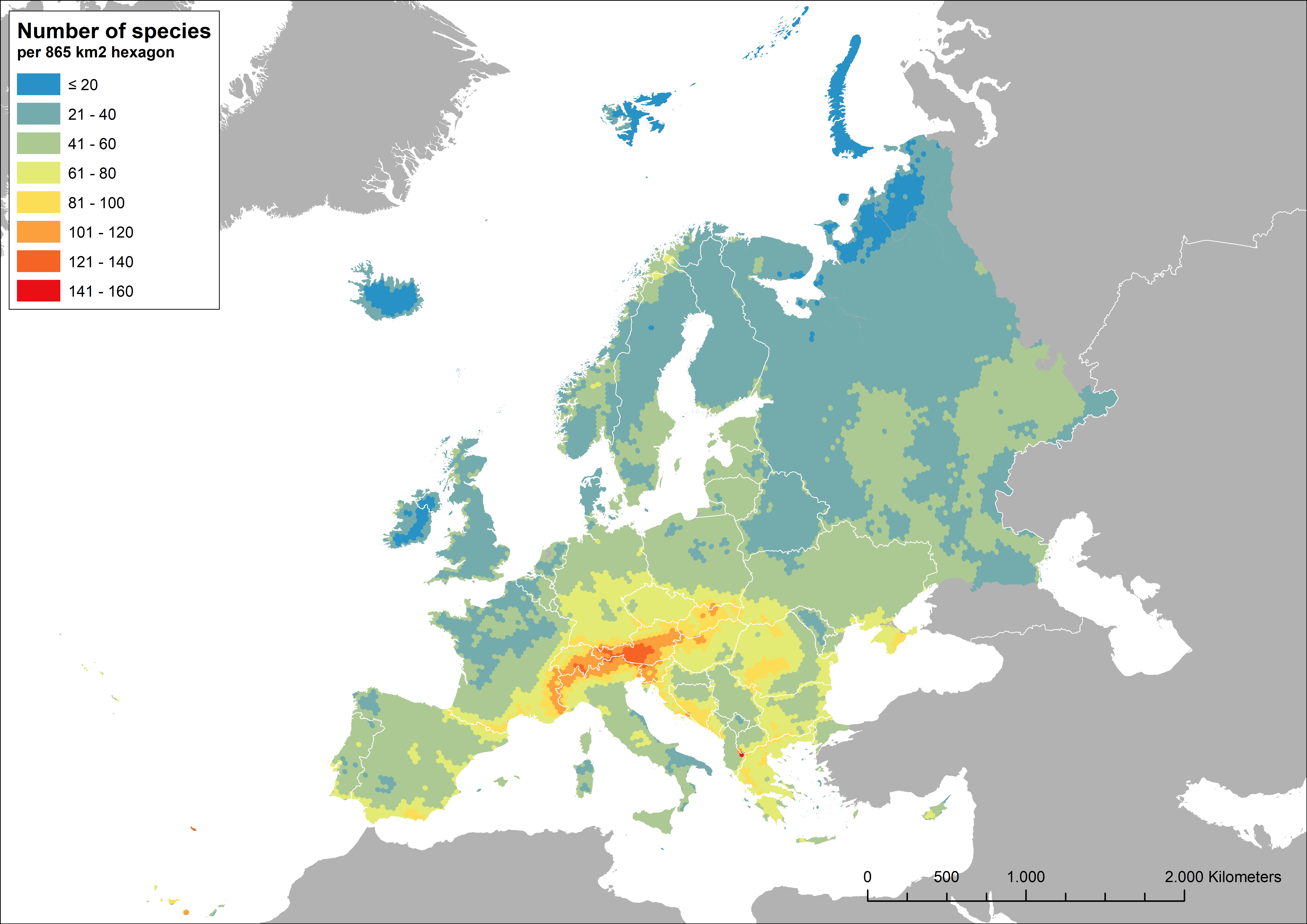

Supplement: S5 Fig — (JPG) [file pone.0293083.s005.jpg]
